# Individual variation and intraclass correlation in arachidonic acid and eicosapentaenoic acid in chicken muscle

**DOI:** 10.1186/1476-511X-9-37

**Published:** 2010-04-15

**Authors:** Anna Haug, Ingrid Olesen, Olav A Christophersen

**Affiliations:** 1 Department of Animal and Aquacultural Sciences, Norwegian University of Life Sciences, N-1432 Ås, Norway; 2 Nofima Marine, P.O.Box 5010, N-1432 Ås, Norway; 3 Ragnhild Schibbyesvei 26, N-0968 Oslo, Norway

## Abstract

Chicken meat with reduced concentration of arachidonic acid (AA) and reduced ratio between *omega*-6 and *omega*-3 fatty acids has potential health benefits because a reduction in AA intake dampens prostanoid signaling, and the proportion between *omega*-6 and *omega*-3 fatty acids is too high in our diet. Analyses for fatty acid determination are expensive, and finding the optimal number of analyses to give reliable results is a challenge. The objective of the present study was i) to analyse the intraclass correlation of different fatty acids in five meat samples, of one gram each, within the same chicken thigh, and ii) to study individual variations in the concentrations of a range of fatty acids and the ratio between *omega*-6 and *omega*-3 fatty acid concentrations among fifteen chickens. Fifteen newly hatched broilers were fed a wheat-based diet containing 4% rapeseed oil and 1% linseed oil for three weeks. Five muscle samples from the mid location of the thigh of each chicken were analysed for fatty acid composition. The intraclass correlation (sample correlation within the same animal) was 0.85-0.98 for the ratios of total *omega*-6 to total *omega*-3 fatty acids and of AA to eicosapentaenoic acid (EPA). This indicates that when studying these fatty acid ratios, one sample of one gram per animal is sufficient. However, due to the high individual variation between chicken for these ratios, a relatively high number of animals (minimum 15) are required to obtain a sufficiently high power to reveal significant effects of experimental factors (*e.g*. feeding regimes). The present experiment resulted in meat with a favorable concentration ratio between *omega*-6 and *omega*-3 fatty acids. The AA concentration varied from 1.5 to 2.8 g/100 g total fatty acids in thigh muscle in the fifteen broilers, and the ratio between AA and EPA concentrations ranged from 2.3 to 3.9. These differences among the birds may be due to genetic variance that can be exploited by breeding for lower AA concentration and/or a more favorable AA/EPA ratio to produce meat with health benefits.

## Background

The focus on meat with a composition adjusted to optimize human health and life expectancy, initiates awareness of meat fatty acid composition. Fatty acid analyses are expensive and the number of samples available may sometimes be limited. It is therefore important to know the number of samples and animals needed for planning an experiment with sufficient power to reveal relevant effects of interest. It can be assumed that there will be enhanced focus on the variation in fatty acid composition between individual animals in a standardized environment, and on the potential to breed for a more favourable fatty acid composition.

Chicken meat is commonly regarded as a healthy type of meat; it is popular, and hence the consumption has increased [1]. Chicken meat is lean, protein-rich and rich also in other important nutrients. However, the fatty acid composition is strongly dependent on the diet fed to the birds. A typical modern poultry diet is rich in cereals having a high ratio between *omega*-6 and *omega*-3 fatty acids. This diet is very different from the natural diet for the same species containing more green leaves that are rich in the *omega*-3 fatty acid *alpha*-linolenic acid (ALA). It has been shown that a diet rich in ALA gives increased concentrations of ALA, eicosapentaenoic acid (EPA), docosapentaenoic acid (DPA) and docosahexaenoic acid (DHA) in broiler muscle and improved, *i.e*. reduced ratio between total *omega*-6 and total *omega*-3 fatty acids [2,3]. The utilisation of ALA and linoleic acid (LA) for synthesizing EPA and arachidonic acid (AA) depends on feed concentrations of ALA and LA as well as on other factors. Much AA in the diet may contribute to prostaglandin overproduction in disease situations in humans, but some AA is necessary for virtually every body function. Dietary sources of AA are especially meat, eggs and offal, with smaller amounts coming from milk and fish.

*Omega*-6 and *omega*-3 fatty acids compete with each other for incorporation into membrane lipids and also for binding to several enzymes such as elongases, desaturases, cyclooxygenases and lipoxygenases [4,5]. *Omega*-3 fatty acids also suppress the expression of inflammatory genes, whereas *omega*-6 fatty acids have an opposite effect [5]. Inflammation takes place within the vascular walls and plays a role in modulating the effect of insulin and control of inflammatory gene expression and lipid metabolism [5]; it is therefore important not only in connection with diabetes type 2, but also as a part of the disease mechanism during progression of atheromatosis/atherosclerosis [5]. *Omega*-3 fatty acids decrease the endothelial responsiveness to proinflammatory and proatherogenic stimuli by modulating the expression of adhesion molecules and cytokines important for the processes collectively denoted as "endothelial activation" [6]. Because the magnitude of postprandial inflammation (which is an independent risk factor for diseases such as atherosclerosis and insulin resistance) depends on the dietary ratio between *omega*-6 and *omega*-3 fatty acids, and because each meal triggers an inflammatory response, this ratio is also an important determinant of the magnitude of the postprandial inflammatory response [7]. In addition, the ratio between *omega*-6 and *omega*-3 fatty acids also influences several processes at the cellular level including rates of cell growth and proliferation, and apoptosis versus cell survival [5].

In a recent study of the substrate specificities of enzymes and prostanoid receptors, it was found that COX-1 oxygenated EPA with only 10% of the efficiency of AA [8]. It was also found that EPA is a comparatively poor inhibitor of AA oxygenation by COX-2. It can be predicted from these and other observations that decreasing the AA/EPA ratios in phospholipids of human cells will reduce the production of 2-series prostanoids and dampen prostanoid signalling. Because AA competes with EPA and DHA as well as with LA, ALA and oleic acid for incorporation in membrane lipids at the same positions, all these fatty acids are important for controlling the AA concentration in membrane lipids, which in turn determines how much AA can be liberated and become available for prostaglandin biosynthesis following phospholipase activation. Thus, the best strategy for dampening prostanoid overproduction in disease situations would be to reduce the intake of AA, or reduce the intake of AA at the same time as the total intake of competing fatty acids (including oleic acid) is enhanced, rather than enhancing intakes of EPA and DHA only. Enhancement of membrane concentrations of EPA and DHA will not be as efficient as a similar decrease in the AA concentration for avoiding prostanoid overproduction.

Combining reduction of the intake of AA with enhancement of the intake of oleic acid will, moreover, also be a better strategy for reducing the total extent of *in vivo *lipid peroxidation, rather than adding more EPA (with 5 double bonds) and DHA (with 6 double bonds) to a diet already over-abundant in arachidonic acid and linoleic acid. A reduction of the dietary ratio of total polyunsaturated fatty acids to oleic acid will not only make plasma lipoproteins less vulnerable to oxidation [9,10], but must also be expected to lead to reduction of the rate of formation of mutagenic aldehydes that arise as secondary products of lipid peroxidation, such as malondialdehyde, crotonaldehyde, acrolein and 4-hydroxynonenal [11]. High rates of production of these mutagenic aldehydes must be expected simultaneously to lead to enhancement of the risk of various forms of cancer (11), and enhancement of the rate of mitochondrial DNA aging, which could lead to earlier onset of various age-associated degenerative diseases perhaps including type 2 diabetes. The degree of fatty acid unsaturation of mitochondrial membrane lipids has been found to be one of those biochemical parameters that are most strongly correlated with longevity, when different species of mammals and birds are compared, with a low degree of fatty unsaturation being correlated with less lipid peroxidation and a longer normal life-span [12]. Oxidatively modified LDL is much more atherogenic than non-modified LDL [13]. Oleic acid has, moreover, also been reported to have antiatherogenic protective effects on endothelial cells by reducing rates of intracellular generation of reactive oxygen species (ROS) [14] and counteracting the activation of nuclear factor-*kappa*B [15].

On the other hand, it is also important that the intake of long-chain *omega*-3 fatty acids should not be too low, given the specific protective roles of these fatty acids against sudden cardiac death [16], and the important role of DHA as a structural component of membrane lipids in the brain [17].

The ratio between concentrations of *omega*-6 and *omega*-3 fatty acids, especially the AA/(EPA + DHA) ratio, is too high in the western diet, and a lower ratio between *omega*-6 to *omega*-3 fatty acids is desirable because it may help to reduce the prevalence and/or morbidity of chronic inflammatory diseases and atherosclerosis [18]. Therefore, we think that all chicken meat available for human consumption should have a favourable ratio between *omega*-6 and *omega*-3 fatty acids, especially when considering the long-chain ones, such as AA, EPA and DHA, and a reduction in the concentration of AA in animal products such as meat, eggs and offal is desirable. At the same time, it would probably also be an advantage if the ratio of oleic acid to sum polyunsaturated fatty acids (especially the oleic acid acid/LA ratio) in these products could be even higher than it is now. This would be expected to improve the storage stability for chicken meat and offal in contact with air (not being stored under inert gas), if they shall contain much more EPA and DHA than now.

Since the sources of the long-chain *omega*-3 fatty acids EPA, DPA and DHA seem to be limited because of ecological limits of total fish production in the sea and a tendency for overfishing of many important fish stocks [19], every step towards increasing the concentration of these fatty acids in the regular human diet from sources other than seafood is of importance.

Chicken meat with a low AA concentration and a favourable ratio between *omega*-6 and *omega*-3 fatty acids may be desirable not only for prophylactic reasons (in order to reduce morbidity and mortality from several important diseases in the general population), but also for making it easier to optimize the composition of the total diet for patients suffering from serious diseases such as cancer, coronary heart disease, rheumatoid arthritis or HIV disease (with the aim of reducing the production of eicosanoids that can be harmful for patients suffering from these diseases, rather than relying on drugs - with their side effects - alone for achieving the same). The same would also be the case for patients suffering from various common forms of pain, *e.g*. because of skeletomuscular disorders.

Increasing the long-chain *omega*-3 fatty acids EPA and DHA intake through the ordinary diet is also a better strategy than relying on fish oil capsules or fish alone, especially when one wishes to reach the entire population. Intake through ordinary foods will be associated with less risk of EPA and DHA peroxidation during storage, compared with fish oil capsules (since rancidification of foods is very easily detected by consumers and an important reason for not eating the food, while the same may not be the case for fish oil inside a capsule). Also when EPA and DHA come from animal foods rather than as purified dietary supplements, they will be ingested together with antioxidant nutrients that are important for prevention of peroxidation *in vivo*, such as selenium, glutathione (plus glutathione precursor amino acids), carnosine and taurine. These antioxidant nutrients (with antioxidant properties protecting against tissue damage caused by ischemia and reperfusion) may themselves have important antimutagenic, anticarcinogenic, and anti-inflammatory properties and may very likely synergize with many of the protective effects of long-chain *omega*-3 fatty acids.

The purpose of the present study was i) to analyse the intraclass correlation of different fatty acids in five meat samples weighing one gram within the same chicken thigh, and ii) to study individual variations in the concentrations of a range of fatty acids and the ratio between *omega*-6 and *omega*-3 fatty acid concentrations among 15 chickens. This ratio may not only depend on the fatty acid composition of the feed, but also on genetic factors, thus opening for an opportunity of breeding for animals with less AA and better *omega*-6/*omega*-3 ratio in the meat.

## Materials and methods

### Animal care

The experimental research on animals followed internationally recognized guidelines. All animals were cared for according to laws and regulations controlling experiments with live animals in Norway (The Animal Protection Act of December 20th, 1974, and the Animal Protection Ordinance Concerning Experiments with Animals of January 15th, 1996), according to the rules given by Norwegian Animal Research Authority.

### Feeding experiment

Fifteen newly hatched male broilers with different unknown dams and sire(s) (Ross 308, Samvirkekylling, Norway) were fed a wheat based diet. During the first 10 days the birds were kept in a deep littered pen; 75 cm × 150 cm. At day 10, each individual was weighed and the birds were placed in separate metabolism cages. They were raised in wire-floored battery brooders in an environmentally controlled room with electrical heating. The room temperature was maintained at 32°C from days 0-3, and then gradually reduced by 0.5°C per day until day 21. The chicks were exposed to 23 h light and 1 h dark photoperiod during days 0-7. The next two weeks they were exposed to 2 × 4 h dark; the dark periods were between 17-21 h and 00-04 h. The birds had free access to water. Water troughs were cleaned daily. At day 21, the birds were weighed and killed by exposure to CO_2 _gas for 2 min in a flow-through system. One thigh was immediately sampled from each bird and frozen for fatty acid composition analyses.

### Experimental diet

#### Diet formulation

The diet included wheat (60%), soybean meal (20%), fish meal (3%), rapeseed oil (4%, Askim Bær- og fruktpresseri, Askim, Norway), linseed oil (1%, Dr. Lindbergs, Oslo, Norway), and nutrient supplements (A/S Norsk Mineralnæring, Hønefoss, Norway). Organic selenium enriched yeast (BioLogics, Ultra Bio-Logics Inc. New O.S.Y. 2000X, containing 2.15 g Se/kg) was added to the diet, giving 0.78 mg/kg diet. Diet composition was 60% Wheat, 20% Soybean meal, 3% Fish meal, 4% Rapeseed oil, 1% Linseed oil, 5% D-fat, 2% Calcium phosphate, 1.85% Ground limestone, 0.25% Sodium chloride, 0.40% DL-Methionine, 0.30% L-Lysine, 0.10% L-Threonine, 0.13% Choline chloride, 0.007% Manganese oxide, 0.04% Se-enriched yeast, 0.15% Mineral mixture, 0.08% Vitamin mixture, (the vitamin and mineral mixture provided per kg diet: retinyl acetate, 2.7 mg; cholecalciferol, 0.07 mg; DL-alpha tocopheryl acetate, 38 mg; menadione, 2.25 mg; pyridoxine, 3.4 mg; riboflavin, 9 mg; Ca-pantothenate, 12.5 mg; biotin, 0.19 mg; thiamine, 1.9 mg; niacin, 37.5 mg; cobalamine, 0.02 mg; folic acid, 1.5 mg; choline chloride, 500 mg; manganese, 75 mg; zinc, 75 mg; iron, 95 mg; copper, 10 mg; iodine, 0.6 mg).

The wheat was ground on a hammer mill, 3 mm sieve. The feed was manufactured at ForTek, Ås, Norway, with matrix type 2.5 m m × 46 m m. 600 kg/hour, cold pelletation.

*The fatty acid concentration *of the diet (g/100 g fat) were: C16:0, 14.3; C18:0, 8.3; C18:1, c9, 37.1; C18:2, n-6, 19.8; C18:3, n-3, 9.0; C20:4, n-6, 0.09; C20:5, n-3, 0.17; C22:5, n-3, 0.06; C22:6, n-3, 0.29.

### Laboratory analyses

#### Fatty acid analyses and determination of fat percent in muscle

Fatty acid composition of five parallel samples, each weighing about one gram from the middle of the thigh from each of the fifteen individuals was determined by gas chromatography. Lipid extraction and direct methylation was performed according to O'Fallon et al. on muscle tissue and feed [20]. Subsequently, the fatty acid methyl esters were analyzed with a 6890N GC with a split/splitless injector, a 7683B automatic liquid sampler, and flame ionization detection (Agilent Technologies, Palo Alto, CA). Separation was performed with a CP-SELECT CB FOR FAME (200 m × 0.25 mm i.d. × 0.25 μm film thickness) fused silica capillary column (Varian Inc.). Temperature program, initial: 70°C with 4 min hold, ramp 20°C/min to 160°C, 3°C/min to 230°C with 15 min hold. The carrier gas was H_2_, and a pressure of 309.4 kPa was used. Fatty acid analysis was performed by autoinjection of 1 μL of each sample at a split ratio of 30:1, a H_2 _flow of 68.4 ml/min and a temperature of 280°C. The flame ionization detector temperature was 290°C with H_2_, air and N_2 _make-up gas flow rates of 40, 450 and 45 ml/min respectively. The sampling frequency was 10 Hz. The run time for a single sample was 91.83 min. Fatty acid peaks determined by gas chromatography were then used to calculate the amounts of fatty acids (g/100 g fat) by theoretical response factors [21]. Considering minoritary fatty acids the "noise" in the baseline of the GC analyses may contribute relatively more to intraassay variability. Standard fatty acids of known composition were run to identify the fatty acids in the samples. Muscle control samples were extracted, methylated and analysed by every 10^th ^sample.

#### Determination of fat percentage in muscle

The percentage of fat was determined in thigh muscle by ASE, Dionex, USA, application note 329, Dionex.

### Statistical Analyses

A one-way ANOVA was carried out for estimating the animal and residual variance components of concentrations and ratios of the fatty acids in the muscle samples using the GLM procedure of the SAS software [22]. The following model was assumed:

where yij is the fatty acid observation of the jth sample of the ith individual, μ is overall mean, ai is the random effect of the ith animal and eij is the random residual error for the jth sample of the ith individual. For each fatty acid character the intraclass correlation was estimated as:

where σa2 and σe2 are estimated animal and residual variance components, respectively.

The correlation coefficients were estimated by the correlation function in Microsoft Excel.

## Results and discussion

Final body weight of the broilers (21 days old when slaughtered) was 928 g, which is as expected for 21 days old broilers, and similar to previous studies [3,23]. None of the birds died during the experiment. The mean fat percent in thigh muscle was 2.4 g/100 g muscle.

### Mean concentrations of fatty acids in the 15 broiler thighs

The mean concentrations of the various fatty acids in broiler thigh in the 15 birds and the standard deviation (SD) are shown in Table 1. The main fatty acids in broiler thigh were oleic acid, palmitic acid, LA and stearic acid (Table 1). *alpha*-Linolenic acid was also a relatively abundant fatty acid. The results are comparable to a previous study [3]. The ratios between the *omega*-6 and *omega*-3 fatty acids (n-6/n-3 and AA/EPA) are also shown in Table 1. The ratio between *omega*-6 and *omega*-3 fatty acids in chicken thigh is reported to be in the range 8-13:1 [24,25]. In the present study, with experimental diets containing 4% rapeseed oil and 1% linseed oil, the broiler thigh meat had a much lower and more favorable ratio between total *omega*-6 and *omega*-3 fatty acids; being about 2:1, and the ratio between AA and EPA was about 3:1. The ratio between AA and EPA was 2.3:1 in the bird with the lowest ratio and 3.9:1 in the bird with the highest ratio, a difference in about 70% which shows that there is a significant variation among the individuals. It is also interesting to note the concentration of AA varying from 1.5 to 2.8 g/100 g fatty acids in thigh muscle in the fifteen broilers. Because the feed composition was the same, it is a reasonable assumption, or working hypothesis, that this variation may have genetic causes.

**Table 1:** Fatty acid composition (g/100 g fatty acids) of chicken thigh muscle.

	Mean	SD	r_e_
C14:0	0.66	0.053	0.49
C16:0	16.03	0.74	0.93
C16:1 c9	2.46	0.47	0.71
C18:0	8.91	0.74	0.50
C18:1 c9	32.98	2.02	0.30
C18:2 n-6, LA	15.45	0.93	0.92
C18:3 n-3, ALA	4.61	0.49	0.43
C20:4 n-6, AA	2.34	0.42	0.34
C20:5 n-3, EPA	0.84	0.15	0.37
C22:5 n-3, DPA	1.58	0.31	0.37
C22:6 n-3, DHA	1.63	0.35	0.40
AA/EPA	2.81	0.46	0.98
n-6/n-3	2.15	0.10	0.85

Mean, standard deviation (SD) and intraclass correlation (r_e_) (n = 15 animals × 5 samples per animal).

This could open for the possibility to breed for chicken that for a given diet composition will have a lower concentration of AA and a lower EPA/DHA ratio in their meat - in which there seems to be a big potential to make the chicken meat even better for the health of the consumer.

### Intraclass correlations and individual variance between birds

Estimated intraclass correlations and between bird variance components are shown in Table 1. For two of the fatty acids; palmitic acid (16:0), and linoleic acid (LA) the intraclass correlation was very high. The ratio between AA and EPA and between total *omega*-6 and total *omega*-3 fatty acids also shows high intraclass correlation, demonstrating that only one sample, weighing one gram, from each thigh and individual will be sufficient for studying these characteristics as very little can be gained by adding an extra sample. However, due to the high variation between individual animals, about 15 animals should be sampled in each group in order to compare different group means with respect to these ratios of fatty acids.

For myristic acid (14:0), stearic acid (18:0) and ALA (18:3 n-3) the intraclass correlation was moderate, but for the fatty acids found in low concentrations; EPA, DHA, DPA and AA, and for oleic acid (18:1 c 9) the intraclass correlation was lower. For these fatty acids, adding an extra sample for analyses from each individual thigh will contribute to increase the power of an experimental study considerably, especially when number of animals per experimental group is low. For example, for oleic acid with intraclass correlation of 0.3, the power of a statistical test (chance that a real difference of 10% is within a 95% confidence interval and hence revealed) of a comparison of two experimental groups of 5 animals increases from 0.74 to 0.90 and 0.95 by adding one or two extra sample(s) per thigh, respectively. Hence, the number of animals in the experiment can be kept low. If only one sample is taken from each animal, we will need 8-10 animals to obtain the same power. This is contrary to *e.g*. LA with an intraclass correlation of 0.9. Here, very little is gained by adding one or 20 extra sample(s) as *e.g*. power increase from 0.80 to 0.82 and 0.84, respectively, when sampling 5 animals per group. Hence, due to the large between animal variance, number of experimental animals per group has to be increased to 7 to reach a power of 0.9.

In a previous study where chickens were fed either a diet containing 4% rapeseed oil plus 1% linseed oil (as the present study) or a diet containing 5% rapeseed oil, the fatty acid composition in thigh muscles were compared. The difference in mean concentration of LA between the dietary treatment groups was less than 10% (15.5 g/100 g fat and 16.4 g/100 g fat) but the difference between the groups was highly significant (P = 0.001) [3].

In order to perform a test of principle we used example data (unpublished data) of two groups of chicken, each of 15 animals, fed either a diet containing 4% rapeseed oil and 1% linseed oil, or a diet containing 5% rapeseed oil. The mean concentration of oleic acid, AA, EPA and DPA in breast muscle differed on average with 19% (10-47%) between the groups, which was significant (P < 0.025-0.0001). When splitting the 15 animals per group randomly into three subgroups of 5 animals per subgroup, only 42% of the 12 t-tests (4 fatty acids × 3 subgroups/data) for the dietary treatments were able to reveal significant differences (P < 0.05). For compatible t-tests of dietary treatments for LA and the ratios AA/EPA and n-6/n-3, showing 4%, 47% and 66% differences, respectively (on average 29% difference), 67% of the nine t-tests (3 fatty acid or ratios × 3 subgroups/data) were significant (P < 0.05). When increasing number of chickens per group to seven and eight per subgroup, 63% of the eight t-tests (3 fatty acids × 2 subgroups/data) of the low intraclass correlated fatty acids (oleic acid, AA, EPA and DPA) were significant. The compatible percentage for the high intraclass correlated fatty acid and ratios (LA, AA/EPA and n-6/n-3) increased to 83%, showing a slightly lower increase in power by increasing number of animals compared to the low intraclass correlated fatty acids. The level of mean differences of the fatty acids varied between the low and high intraclass correlated fatty acids (29% versus 19%), but the large differences were accompanied with very low standard errors, yielding a high level of significance (P < 0.0021 for five of the six significant t-tests). For the low intraclass correlated fatty acids, the results showed that tests of dietary effects based on five animals per diet are very poor, and that increasing number of animals from five to seven or eight animals will give much more powerful tests.

### Correlation coefficients

The associations (correlation coefficients) between various fatty acids are shown in Table 2, showing that the concentration of the dominant fatty acid in broiler muscle, oleic acid (33% of the fatty acid methyl esters) is strongly and positively correlated to those of the monounsaturated fatty acids 16:1 c9 and also to 14:1 c9 (not shown in table). These are all fatty acids produced by desaturation with the *delta*-9 desaturase enzyme. There is also a strong but negative association between oleic acid and stearic acid. This can be explained by the fact that stearic acid is converted to oleic acid by the *delta*-9 desaturase enzyme. Oleic acid also correlates highly negatively to the C20 and C22 fatty acids and positively to the calculated ratios LA to AA, ALA to EPA, ALA to DPA, ALA to DHA and ALA to LA. This suggests that oleic acid might inhibit elongation/desaturation processes either directly (*e.g*. as a competitive inhibitor of linoleoyl-CoA and *alpha*-linolenoyl-CoA formation) or by some indirect mechanism. It has been suggested that conversion of fatty acids to their long-chain highly unsaturated products is stimulated in situations when there is a deficiency of antioxidants [26]. Our observations are compatible with these observations, as oleic acid is a stable fatty acid not triggering oxidative stress. But the hypothesis that oleic acid functions as a competitive inhibitor of LA and ALA conversion into long-chain polyunsaturated fatty acids (perhaps especially at the very first step of the pathways concerned, but quite plausibly also at the next one, *i.e*. that of fatty acyl-CoA elongation), would appear equally plausible.

**Table 2:** Coefficients of correlations between fatty acids and some fatty acid ratios in chicken thigh muscle (n = 15).

	C16:0	C16:1 c9	C18:0	C18:1 c9	C18:2 n6	C18:3 n3	C20:4 n6	C20:5 n3	C22:5 n3	C22:6 n3	AA/EPA	n-6/n-3	LA/AA	ALA/EPA	ALA/DPA	ALA/DHA	ALA/LA
C16:0	1,00																
C16:1 c9	0,56	1,00															
C18:0	-0,18	-0,79	1,00														
C18:1 c9	0,09	**0,73**	-0,86	1,00													
C18:2 n6	-0,83	-0,66	0,26	-0,25	1,00												
C18:3 n3	-0,35	0,27	-0,72	**0,70**	0,38	1,00											
C20:4 n6	-0,40	-0,76	**0,85**	-0,84	0,29	-0,65	1,00										
C20:5 n3	0,26	-0,31	**0,64**	-0,79	-0,24	-0,86	**0,63**	1,00									
C22:5 n3	-0,10	-0,58	**0,73**	-0,89	0,03	-0,73	**0,86**	**0,84**	1,00								
C22:6 n3	-0,07	-0,69	**0,80**	-0,91	0,14	-0,68	**0,85**	**0,63**	**0,82**	1,00							
AA/EPA	-0,78	-0,53	0,24	-0,09	**0,65**	0,23	0,45	-0,40	0,03	0,28	1,00						
n-6/n-3	-0,60	-0,29	0,15	0,14	**0,66**	0,24	0,05	-0,36	-0,34	-0,24	0,50	1,00					
LA/AA	0,14	**0,63**	-0,85	**0,85**	-0,03	**0,82**	-0,95	-0,77	-0,89	-0,86	-0,23	0,13	1,00				
ALA/EPA	-0,30	**0,37**	-0,75	**0,81**	0,26	**0,93**	-0,67	-0,96	-0,82	-0,69	0,33	0,31	**0,83**	1,00			
ALA/DPA	-0,02	**0,57**	-0,81	**0,89**	0,03	**0,85**	-0,84	-0,89	-0,94	-0,81	0,04	0,25	**0,94**	**0,93**	1,00		
ALA/DHA	-0,11	**0,63**	-0,87	**0,89**	0,00	**0,83**	-0,79	-0,73	-0,78	-0,94	-0,10	0,20	**0,88**	**0,83**	**0,87**	1,00	
ALA/LA	0,11	**0,65**	-0,89	**0,89**	-0,16	**0,85**	-0,86	-0,79	-0,81	-0,81	-0,12	-0,10	**0,89**	**0,83**	**0,88**	**0,87**	1,00

LA; Linoleic acid. ALA; alpha-linolenic acid. AA; arachidonic acid. EPA; eicosapentaenoic acid.

DPA; docosapentaenoic acid. DHA; docosahexaenoic acid.

In the same manner as oleic acid, ALA also shows a negative association to the C20 and C22 fatty acids, and positive association to the calculated ratios LA to AA, ALA to EPA, ALA to DPA, ALA to DHA and ALA to LA. This, however, must have a different explanation, because ALA with its three double bonds is not a fatty acid that can contribute to protect membrane lipids against peroxidation, as oleic acid does. We shall return to this question later.

Stearic acid, being negatively correlated to oleic acid, shows a positive association to fatty acids with 20 and 22 C-atoms, both *omega*-3 and *omega*-6, and a negative association to the calculated concentration ratios LA to AA, ALA to EPA, ALA to DPA, ALA to DHA and ALA to LA, indicating that either a high level of stearic acid *per se *or less conversion of stearic acid to oleic acid (functioning as an inhibitor) might favor conversion of LA and ALA into the long 20 and 22 C fatty acids. The C20 and C22 n-6 and n-3 fatty acids are all positively correlated to each other, indicating that they might be controlled in a similar way by the same genetically variable factors (which *e.g*. might be a genetically controlled difference in the expression of one or more enzymes in the pathway of LA and ALA conversion into the corresponding C20 and C22 fatty acids, but also a genetically controlled difference in the rate of conversion of stearic acid into oleic acid).

The observed positive correlation between the *omega*-6 fatty acid AA and the *omega*-3 fatty acids EPA, DPA and DHA might indicate that the genetic regulation of the expression or activity of the elongases and desaturases may covariate with both the conversion of the *omega*-6 fatty acid LA to AA and of the *omega*-3 fatty acid ALA to EPA, DPA and DHA (Table 2). These fatty acids compete in binding to enzymes. However, the positive association between AA and EPA, DPA and DHA shows that the desired reduction in AA will also be followed by a reduction in EPA, DPA and DHA.

The intraclass correlation indicates the maximum level of heritability of a trait. Because the feed composition was identical for all animals, it may be speculated whether some of the individual variation may be due to genetic factors implying differences in the kinetic properties or expression of individual enzymes (elongases or desaturases) that participate in the conversion of LA and ALA to the corresponding long-chain fatty acids. However, there may also be differences in the rate of fatty acid consumption as metabolic fuels.

The strongly negative correlation found between ALA and long-chain *omega*-3 fatty acids could possibly be explained assuming a strong preference for ALA compared to LA at the start of the biochemical pathway of conversion of these fatty acids to the corresponding long-chain fatty acids. Animals with more rapid *beta*-oxidation of C18 polyunsaturated fatty acids might then contain less LA and AA, while animals with more rapid conversion of C18 polyunsaturated fatty acids to corresponding C20 and C22 fatty acids will contain less ALA and more EPA and DHA. It is not unreasonable that there might be a preferential conversion of ALA (rather than LA) conversion into long-chain fatty acids in poultry, given the importance of DHA for the normal function of the central nervous system [27] that is presumably not much different in birds compared with humans and also the high *omega*-6/*omega*-3 fatty acid ratio of many seeds that may be important as food for birds living in their natural habitats.

It is possible that there is a similar preference for ALA, compared to LA, by enzymes of the elongase-desaturase pathway also in humans, as suggested by the much lower ALA/LA concentration ratio in human blood samples from the population in Crete, compared to olive oil [28]. In this study they found that the plasma lipoprotein cholesteryl ester contained 11.1 +/- 1.0 palmitic acid, 31.0 +/- 2.7% oleic acid, 41.9 +/- 3.7% LA and only 0.9 +/- 0.5% ALA. It would appear more plausible to explain the much lower ALA/LA ratio found in human blood cholesteryl esters than in the traditional diet at Crete as a consequence of preferential conversion of ALA into EPA, DPA and DHA, rather than as a consequence of more rapid *beta*-oxidation of ALA compared to LA. This hypothesis implies that there must be a high degree of ALA conversion into long-chain *omega*-3 fatty acids in humans, which is in conflict with research reports suggesting very poor conversion of ALA into EPA and DHA in humans [29,30]. Literature data in this field are not consistent; since some groups [31] have found considerably better conversion than others have done. It may be questioned if this might reflect variations in the intake of some unidentified nutrient cofactor needed for normal conversion of ALA into EPA and DHA. One possible (but entirely hypothetical) candidate is the trace element vanadium, since the bioavailability of this element for uptake into plant roots is heavily dependent on redox conditions in the soil, and it is not unreasonable that the dietary intake of vanadium could be much higher in Crete than in Canada (because of higher soil temperature and lower organic matter concentrations combined with higher average pH in the soils in Crete). This trace metal has also more general chemical properties (redox behaviour, ionic radii and coordination chemistry in crystals and complexes) that could make it potentially useful as a catalyst of fatty acid desaturation reactions, and there are observations from animal experiments suggesting that vanadium deficiency may cause disturbances in lipid metabolism [32].

An alternative hypothesis for explaining our own observations would be to postulate a genetic variance in the substrate specificity (for ALA versus LA) of the enzyme at the start of the pathway of C18 polyunsaturated fatty acid conversion into corresponding C20 polyunsaturated fatty acids. This hypothesis is not implausible from the evolutionary point of view because it could offer a good mechanism of genetic adaptation of animals to different types of environment or ecological niches, where the food available has significantly different overall *omega*-6/*omega*-3 fatty acid ratios.

If such genetic differences in the metabolic conversion of C18 fatty acids into C20 and C22 fatty acids do indeed exist, this could obviously be of health-economic relevance in our society. In the present situation, when poultry is most commonly given a feed with very high LA/ALA ratio, it would be an advantage for the consumer if the animals have a poor conversion of LA into AA. However, if regulations should be imposed by the health authorities in Norway or other countries, requiring that the overall LA/ALA ratio in poultry feed should not be higher than perhaps 2/1, we would rather desire that as much as possible of the ALA in the feed could be converted into EPA and DHA.

Our data indicating genetic variation in fatty acid metabolism in chicken are in partial agreement with earlier reports. De Smet et al. [33] concluded in a review that there may some degree of genetically controlled variation in the concentrations of monounsaturated and saturated fatty acids among individuals after correcting for fat level. However, they suggested that enzyme activities were not able to explain between-animal variation in fatty acid composition [33].

While the most important method for optimizing the fatty acid composition of poultry meat still must be to optimize the fatty acid composition of the feed, we can not exclude the possibility that some improvement could be obtained through breeding efforts. Especially it might be possible to change the substrate specificity of some of the enzymes concerned for more efficient conversion of ALA and less efficient conversion of LA into the corresponding long-chain polyunsaturated fatty acids.

An optimization of the fatty acid composition of the total human diet - not only at the level of individuals, but also at a population level - is important. The proportions between the C18 acids oleic acid, LA and ALA in the total diet are heavily dependent on consumption levels and the fatty acid composition of various edible fats and oils. Intakes of AA, EPA, DPA and DHA are mainly determined by the intake level and fatty acid composition of various fish- and other animal foods, as well as by the consumption of dietary supplements (especially in form of cod-liver oil and fish oil capsules).

A high absolute intake of AA is directly associated with a tendency for prostaglandin and thromboxane overproduction. Thromboxane A_2_, which is a potent vasoconstrictor and platelet aggregator [34], is mainly produced in the platelets, where COX-1 is constitutionally expressed. It was earlier thought that thromboxane A_3 _is inactive. This has now been shown not to be correct [8]. However, the rate of EPA oxidation by COX-1 is only 10% of the rate of AA oxidation [8], which can still help to explain why a high dietary ratio of EPA to AA is antithrombotic, while a high dietary intake of AA enhances the risk of thrombotic events. This will especially be the case when simultaneously a combination of high rates of reactive oxygen species (ROS) production, *e.g*. as a consequence of hyperglycemia or hypertension [35-37], and poor antioxidant defense capacity in blood plasma and the endothelial cells, *e.g*. because of selenium depletion [38-40] or glutathione depletion [14,15,41,42], leads to inhibition of endothelial prostacyclin synthetase by peroxynitrite [43] or fatty acid hydroperoxides [44], so that thromboxane A_2 _production in activated platelets will be high at the same time as prostacyclin production in the endothelium is depressed. Prostacyclins (PGI_2 _and PGI_3_) are at the same time very potent vasodilators and very potent inhibitors of platelet aggregation [34], and it is therefore strongly dependent on the thromboxane/prostacyclin balance whether platelet-initiated or vascular wall-initiated thrombosis will occur or not.

Chicken meat with a low AA/(EPA + DHA) ratio would be expected not only to improve the AA/(EPA + DHA) ratio in membrane lipids both in the endothelium and platelets, but also (and in contrast to fish oil capsules) to counteract glutathione depletion in the same cells, *e.g*. in patients suffering from acute or chronic infectious disease or other protein catabolic conditions - in which case not only the total rate of protein catabolism is enhanced [45], but also the rate of degradation of sulphur amino acids [46,47]. A similar doubly protective effect must also be expected for fat fish products, such as mackerel and herring, and for products made from whole fish, such as dried kapenta and fish protein concentrate type B (FPC type B) [48]. Most fish products have also important antioxidative protective effects because of their high selenium concentration [49,50]. In poultry meat it is possible to achieve a similar selenium/protein concentration ratio as in fish by adding selenomethionine rather than sodium selenite to the feed [3].

Disease situations where COX-2 is important include many (but far from all) cases of cancer, including a substantial proportion of patients with colorectal cancer, breast cancer, lung cancer and prostate cancer [51]. COX-2 is, moreover, also important in various chronic non-infectious inflammatory diseases, such as rheumatoid arthritis [34], and in pain conditions attending skeletomuscular disorders. There are many reasons why it is important to limit prostaglandin production in tumors expressing COX-2. Prostaglandin E_2 _(PGE_2_), which is abundantly produced in such tumors [52], stimulates tumor angiogenesis [52], *i.e*. the ingrowth of blood vessels in the tumor [52,53], which will in turn reduce oxygen and nutrient limitation [54] of the rate of tumor growth, thus leading to faster progression of the disease. PGE_2 _also inhibits NK cells [55], lymphokine-activated killer (LAK) cells [56] and cytotoxic CD8^+ ^cells [57], which all are important in anti-tumor immunological defense [55,58,59], at the same time as prostaglandin overproduction in the tumor also must be expected to increase pain associated with cancer.

There are therefore several good reasons not only to enhance dietary intakes of EPA and DHA, but also to limit the dietary intake of AA in all such cases of cancer where the tumor cells express COX-2. Patients with COX-2-expressing tumor cells should therefore be told to avoid poultry meat, swine meat and egg with high AA/(EPA + DHA) ratios. But all these foods would probably be safe for cancer patients to eat if they had a more natural fatty acid composition than is common in most of the western world (including both North America and Western Europe) today. At the same time, it should not be forgotten that anti-tumor immunological functions - that overlap strongly with NK-cell- and T-cell-mediated functions important for antiviral immunological defense - also depend on glutamine, glutathione and selenium status [60,61]. As an example can be mentioned that NK cells normally will secrete the Th1-associated cytokine interferon-*gamma *following simultaneous stimulation with interleukin-12 (IL-12) and interleukin-2 (IL-2), provided that they are not glutathione-depleted [62]. But glutathione-depleted NK cells will instead secrete the Th2-associated (and Th1-inhibitory) cytokine interleukin-10 (IL-10) following double stimulation with IL-12 and IL-2 [62].

Thus the broiler meat in the present study could be a healthful element of the human diet, since it may not only help to increase the intakes of EPA, DPA and DHA from ordinary foods, but also to decrease the intake of arachidonic acid that is now too high in much of the western world, at the same time as it also functions as a good source of nutrients that are important for antioxidant protection in endothelial cells and platelets (and thus for prevention of atheromatosis and thrombosis), as well as being important for immunological defense both in infectious diseases and cancer.

## Conclusion

We have found that a broiler feed containing 4% rapeseed oil and 1% linseed oil/kg diet gave a meat that was characterized by a favourable ratio between AA and EPA of about 3:1. Moderate to high intraclass correlations (0.30-0.92) were estimated for all the fatty acids analyzed, and particularly high estimates were obtained for the ratios of AA/EPA and n-6/n-3 (0.85-0.98) indicating a potentially high heritability. The ratio between AA and EPA was 2.3:1 in the bird with the lowest ratio and 3.9 :1 in the bird with the highest ratio. The estimated intraclass correlation for AA was much lower (0.34), but still the concentration of AA varied from 1.5 to 2.8 g/100 g fatty acid in thigh muscle in the fifteen broilers. Thus breeding for healthier meat containing low AA concentration and low AA/EPA ratio in chicken meat may be possible. One thigh muscle sample of one gram was enough to give reliable estimates of the AA/EPA ratio and *omega*-6/*omega*-3 ratio of a chicken thigh. For the trustworthy absolute concentration of AA, EPA, DPA and DHA, adding one (or more) sample(s) of one gram thigh meat sample will contribute significantly to reduce standard errors of the estimates, and improve the power of experimental studies, especially when the number of animals per group is low. For the fatty acids and fatty acids ratios with high intraclass correlations (C16:0, LA, AA/EPA and n-6/n-3), only one sample per animal is enough, but higher number of animals (about 15 animals) is needed per experimental group to be compared.

## Competing interests

The authors declare that they have no competing interests.

## Nature of contribution of authors

AH was major contributor in planning, experimental work, analysis and publication of the results. IO contributed in statistical data handling, writing of the article and discussion. OAC contributed in planning of the experiment, discussing the results and writing the article. All authors have read and approved the manuscript.
